# Can’t stand the look in the mirror? Self-awareness avoidance in borderline personality disorder

**DOI:** 10.1186/s40479-015-0034-9

**Published:** 2015-11-14

**Authors:** Dorina Winter, Katrin Koplin, Stefanie Lis

**Affiliations:** Department of Psychosomatic Medicine and Psychotherapy, Central Institute of Mental Health, Medical Faculty Mannheim/Heidelberg University, Mannheim, Germany; Institute for Psychiatric and Psychosomatic Psychotherapy, Central Institute of Mental Health, Medical Faculty Mannheim/Heidelberg University, Mannheim, Germany

**Keywords:** Personality disorders, Self-awareness, Self-reference, Social rejection, Self-focused attention

## Abstract

**Background:**

Patients with Borderline Personality Disorder (BPD) expect and perceive social rejection stronger than healthy individuals. Shifting ones attention from oneself to others has been suggested as a mechanism to deal with the experience of social rejection. Here, we investigated whether BPD participants avoid increased self-awareness and whether this is done intentionally.

**Methods:**

Thirty BPD patients and 30 healthy control participants, all naïve of the study’s purpose, were asked to choose either a seat facing a mirror (self-awareness) or not facing the mirror (avoidance of self-awareness). Afterwards they were asked to indicate if they have chosen the seat intentionally.

**Results:**

BPD patients avoided as a trend the chair facing the mirror more often than healthy control participants. 90 % of the patients reported that they made their seating decision intentionally in contrast to 26.7 % of the healthy participants (odd ratio = 24.75).

**Conclusions:**

Results revealed altered reactions to self-awareness cues in BPD. While BPD patients avoided such a cue slightly more often, they were more often aware of their behavior than healthy participants. As possible explanations, a negative body related, shame-prone self-concept as well as a simultaneously increased degree of self-focused attention are suggested.

## Theoretical background

Enduring difficulties in social interactions, including unstable and intense interpersonal relationships, and frantic efforts to avoid abandonment reflect basic characteristics of borderline personality disorder (BPD; [1–3]). As one factor contributing to these difficulties, studies demonstrated that BPD patients expect to be rejected more frequently in social interactions than healthy controls and are more concerned by this perception in social interactions [4–7]. BPD patients perceived to be more excluded from an interaction even when, objectively, they were included as much as every other team member [6, 8, 9]. This suggests that BPD patients anxiously expect social rejection even in the absence of an acute rejection situation.

One mechanism to deal with perceived rejection has been shown to be a shift in one owns attention from the self to others [10, 11]. The decrease in attention to the self was suggested to protect the self from devaluating experiences, while the increase of attention to others would serve to re-establish and maintain social relationships [10]. Such attention shifts has been measured by simple behavioral tests such as the choice of participants to sit down on a chair in front of a mirror or not [12]. Twenge and colleagues [11] used this paradigm to investigate effects of social rejection induced by faked feedback on a personality questionnaire: Participants, who were told that their questionnaire results show that they are likely to spend their future alone, chose less often the chair facing a mirror compared to those participants without feedback as well as those that were told that the results predict rewarding relationships or misfortune. The authors concluded that people who feel socially excluded are averse to attention to the self. It has been shown that self-awareness initiates self-evaluation [13–16]. Thus, avoiding cues that increase self-awareness may help to avoid or reduce negative self-evaluative processes such as those induced by assuming to have a personality that is so miserable that it will lead to a lonely future [11]. Assuming that BPD patients expect being rejected in the absence of acute social exclusion, they may avoid cues of self-awareness more than healthy control participants without such an exclusion context.

Due to these findings, we hypothesized that BPD patients would avoid to sit on a chair facing to a mirror even in the absence of an actual rejection experience, which healthy control participants would do less often. As self-awareness cues may be particularly salient to BPD patients due to their negative and shame-prone self-concept [17–21], we also expected that BPD patients would adjust their behavior more intentionally. To examine this, we were also interested in whether the seating would be chosen intentionally or not and whether this would differ between BPD patients and healthy controls.

## Methods

### Sample

Thirty women with BPD and 30 healthy women (HC) matched according to age and education participated in this study as part of a larger study on self-referential processing. We informed all participants regarding study procedures and obtained written informed consent. The study followed the Declaration of Helsinki. Research Ethics Board II of Heidelberg University, Germany, had approved the study. Exclusion criteria for all participants were any traumatic brain injuries and lifetime schizophrenia or bipolar I disorder, mental or developmental disorders as well as substance dependency during the last year and current substance abuse. HC had to be free of any current or lifetime mental illness and psychotropic medication. Trained clinical psychologist obtained the diagnosis of BPD using the International Personality Disorder Examination (IPDE; [22]). Axis I disorders were assessed using the Structured Interview for DSM-IV [23]. Borderline symptom severity was measured using the short version of the Borderline Symptom List (BSL-23; [24]).

Sample characteristics are presented in Table 1. BPD patients and healthy controls did not differ in age and education. 19 (63.3 %) of the BPD patients were free of psychotropic medication, 6 (20 %) received an atypical antipsychotic, 5 (16.7 %) selective serotonin reuptake inhibitors, 4 (13.3 %) serotonin-norepinephrine reuptake inhibitors, and one (3.3 %) each monoamine oxidase inhibitors, tetracyclic antidepressants, neuroleptic medication, and methylphenidate.

**Table 1:** Demographic and clinical variables in healthy control participants (HC) and patients with Borderline Personality Disorder (BPD)

	HC (*n* = 30)		BPD (*n* = 30)		Statistics	
	*AM*	*SD* (±)	*AM*	*SD* (±)	*T*	*P*
Age - years	26.13	7.29	26.10	4.76	*t* = 0.21	.983
Years of education, *n (%)*						
9 years	0	(0)	4	(13.33)	*U* = 409	.492
10 years	13	(43.33)	10	(33.33)	*Z* = −0.69	
13 years	17	(46.67)	16	(53.33)		
Borderline Symptom List-23 (mean)	0.10	0.15	2.42	0.71	*t* = −17.55	<.001
Rejection sensitivity questionnaire^a^	6.2	2.9	16.8	6.2	*t* = −7.94	<.001
Co-morbidities, *n (%)*						
major depressive disorder			2	(6.67)		
dysthymia			2	(6.67)		
panic disorder with agoraphobia			2	(6.67)		
social phobia			8	(26.67)		
specific phobia			2	(6.67)		
obsessive compulsive disorder			2	(6.67)		
posttraumatic stress disorder			17	(56.67)		
somatization disorder			1	(3.33)		
unspecific somatoform disorder			2	(6.67)		
bulimia nervosa			2	(6.67)		
binge eating disorder			5	(16.67)		
dissociative convulsions			1	(3.33)		

^a^data of 2 HC and 4 BPD was not evaluable; AM = arithmetic mean; SD = standard deviation

### Experimental task

**Fig. 1:**
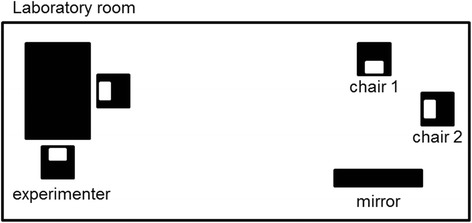
Experimental set up. Participants were supposed to choose either the chair facing the mirror (increased self-awareness, chair 1) or the chair facing the room (self-awareness avoidance, chair 2)

After participants completed the study described in [25], participants were told that the experiment was over but that there were some final diagnostic questions that the experimenter would like to ask. That would take place in another corner of the laboratory room. There were two chairs standing with their back to a wall, one facing a standardized (= distortion-free) mirror (height 175 cm, width 95 cm) and one facing into the room. See Fig. 1 for illustration. The experimenter asked the participant to sit down while she would save some data and would join the participant afterwards. In case the participant asked which chair she should sit down on, the experimenter told her to feel free to sit down on any chair she prefers. The instructions were standardized in order to avoid an experimenter bias. The experimenter did not engage in any further verbal or nonverbal interaction with the participant at this stage of the experiment. Immediately after the participant sat down on one of the two chairs, the experimenter recorded the participant’s choice and asked whether it was made intentionally.

### Statistical analysis

The dependent variables were the number of participants choosing the chair not facing the mirror (behavioral avoidance) as well as the number of participants choosing their seating position intentionally (choice intentionality) per group (HC, BPD). Whether more BPD patients displayed behavioral avoidance or an intentional decision than healthy control participants was tested using Fisher’s exact tests (one-tailed for behavioral avoidance, two-tailed for choice intentionality). All analyses were performed with IBM SPSS Statistics 20 (IBM, USA).

## Results

With respect to *behavioral avoidance,* 21 (70 %) healthy control participants and 27 (90 %) BPD patients did choose the chair not facing the mirror. Statistical analysis revealed a strong trend that more BPD patients than HC avoided the chair facing the mirror (*p* = .052, *OR* = 3.98).

**Fig. 2:**
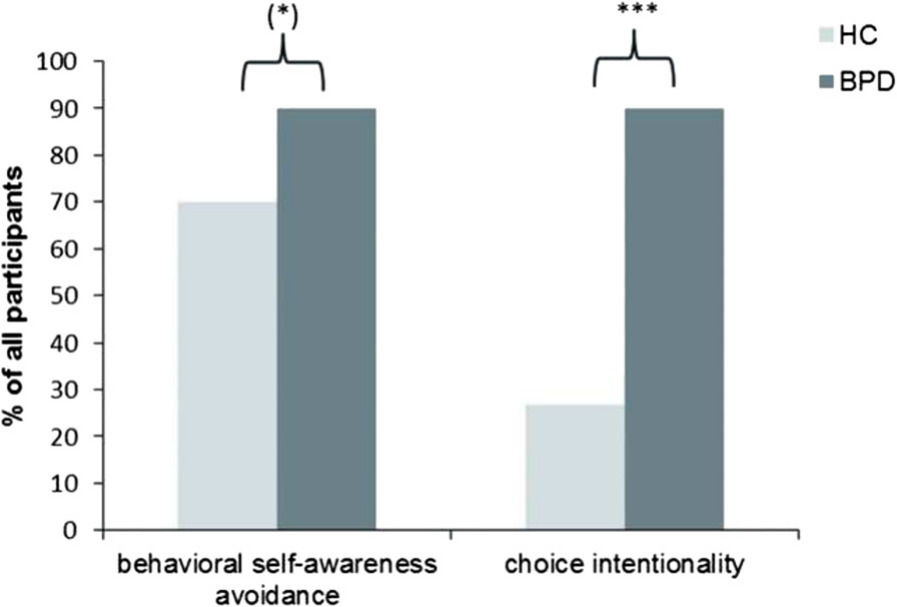
Behavioral Results. Results on the behavioral self-awareness avoidance reflected by the percentages of subjects choosing the chair not facing the mirror and the reported intentionality of this choice. BPD = borderline personality disorder, HC = healthy control participants

Regarding *choice intentionality,* more BPD patients (*N* = 27, 90.0 %) than HC (*N* = 8, 26.7 %) reported to have chosen the chair intentionally (*p* < .001, *OR* = 24.75). See Fig. 2 for graphical illustration. To explore whether the chosen seating influenced the choice intentionality, we compared the choice intentionality for those participants facing and not facing the mirror. Of the 21 HCs avoiding the mirror image, 28.6 % (*N* = 6) reported an intentional choice, which was similar to those facing the mirror (22.2 %, *N* = 2 out of *N* = 9, *p* = 1.00). Of the 27 BPD patients avoiding the mirror image, 92.6 % (*N* = 25) reported an intentional choice, while this was true for 66.6 % of those facing the mirror (*N* = 2 out of *N* = 3). The small sample size of BPD patients facing the mirror prevents a further statistical analysis and an investigation of modulating factors. However, it seems worth mentioning that those three patients who reported a non-consciousness seating choice reported lower RSQ-scores (9.5 *SD* 1.1) than 84.6 % of the BPD sample.

## Discussion

This study investigated reactions to self-awareness cues in BPD. Since self-awareness avoidance has been described in healthy individuals as a response to social exclusion [10, 11, 26], we hypothesized that BPD patients would avoid self-awareness even in the absence of actual social exclusion. In addition, we were interested in whether participants were aware of avoidance behavior and whether this differs between HC and BPD suggesting an altered salience of self-related processes in BPD. Our findings mainly confirmed our hypothesis: BPD patients avoided the chair facing the mirror slightly more often than healthy controls. However, in contrast to this statistically only marginally significant finding, groups differed strongly in regard to the intentionality of their choices: BPD patients indicated more often than healthy controls that they have been conscious about their seating decision.

A heightened tendency not to sit down on a chair facing a mirror was proposed to reflect self-awareness avoidance [11]. According to previous evidence [10, 11], healthy individuals would allocate their attention away from themselves and towards social others after acute rejection, probably in order to increase the awareness of social signals of a partner to re-establish a social relationship. Even without experimental rejection induction, BPD patients avoided cues that increase attention to the self more often than HC. The high proportion of BPD patients that chose the chair not facing the mirror is comparable to that in HC after being acutely socially rejected (see [11]).

In the present study, a high percentage of HCs chose the chair not facing the mirror without the experimental induced experience of social rejection. This proportion of HC that avoided cues of self-awareness is similar to that found by Twenge et al. [11] when participants were facing socially relevant positive information. An explanation might be that healthy participants shifted their awareness away from their physical appearance in preparation of the announced, subsequent conversation and social interaction.

With respect to their seating choice, BPD patients were in contrast to HC well aware of their behaviour: While only 27 % of the HC reported to have chosen the seating intentionally, about 90 % of the patients reported to have been aware of their decision for one of the chairs. Such particularly high self-focused attention has been found to play a role in the maintenance of social anxiety [27–29]. Clark and Wells [27] stress that socially anxious people monitor themselves as ‘social objects’, which - due to negative assumptions about themselves - leads to heightened perception of social threat with respective somatic and cognitive symptoms as well as safety behaviours such as escape from a social situation. When self-awareness is increased using e.g. a video-camera [30–32], anxiety and escape propensity also increases - a phenomenon also observed in individuals with low self-esteem [33]. This is in agreement with clinical observations that BPD patients react with an increase of inner tension in situations that require public speech, videotaping or body related interventions. Similarly, patients with posttraumatic stress disorder after childhood sexual abuse, a frequent comorbidity in BPD, experienced increased inner tension when anticipating a mirror confrontation, i.e. when being asked to focus at different parts of their own body while wearing a standard bikini [34].

Taken together, our findings revealed complex alterations of self-awareness in BPD: While behaviourally avoiding cues that direct attention to the self, the conscious seating choice suggests an increased self-awareness probably linked to self-evaluative processes in BPD. One may ask whether the study’s results on altered self-awareness in BPD can actually be linked to a history of social rejection in BPD. Our findings are similarly conclusive with earlier findings of maladaptive perception of one’s own body in BPD [18, 35–37]. BPD patients have a negative self-concept comprising a negative body image [17–21, 35, 38]. They have been shown to have a negative attitude towards their physical self and they have reported to avoid social situations during which physical appearance may be important [35]. Similarly, comorbid BPD increased the attention to body related cues in posttraumatic stress disorder although this attentional bias was not explicitly linked to the own body [39]. Future studies have to investigate the interplay between a negative body image and fear of social rejection. One may speculate whether a negative body image constitutes a threat to social participation and belonging: particularly when physical appearance is assessed as important, social rejection may be anticipated when the own appearance is negatively evaluated. Another important factor might be the experience of shame. Shame is a self-conscious emotion associated with a negative body image and plays an essential role in the self-concept of BPD patients [17–21]. It has been linked to increased self-awareness [40]. Similar to the relation between negative body image and shame, shame may also be a key emotional response to social threats such as social exclusion [41]. In BPD, studies on the relation of shame and social rejection are so far inconclusive: While Chapman et al. [42] observed an increase of shame particularly after the experience of social rejection, a subsequent study could not replicate this finding [43]. Taken together, experienced and expected rejection, a negative body image, and shame may contribute to our findings in BPD, showing the attempt to reduce body-related self-awareness by avoiding self-related cues while, however, simultaneously failing to withdraw attention from the own behavior and reduce self-awareness. Since we did not measure shame and body image in our sample, further studies are required to disentangle the relevance of experienced or imagined social rejection, a negative body image and shame for alterations of self-awareness in BPD. It has to be emphasized that subsequent studies have to apply finer-grained measurement variables providing a higher variance between participants to allow for the identification of modulating factors. Additionally, alternative measurement methods of self-awareness have to be applied (see, e.g., sentence completion tasks with first-person pronouns [44]) to test whether the alterations of self-awareness in BPD are restricted to body-related cues or extend to other features of the self.

Regarding this study, some limitations need to be considered. We only included female participants, thus the study does not allow any conclusions regarding male BPD patients. It has to be mentioned that the sample size is small and further independent studies have to replicate our findings. Future studies may aim at including a higher number of patients to allow for an analysis of specific traits or comorbidities to gain further insight into the underlying mechanism of the described self-awareness alterations. To assess the specificity of our findings for BPD, future studies with clinical control groups such as patients with social phobia, PTSD and other personality disorders are required. Based on our data, it is not possible to decide whether behavioural avoidance and the intentionality of this strategy represent an unspecific mechanism linked to psychopathology in general, to people low in self-esteem or high in social anxiety or whether they are specific to BPD. Nevertheless, our findings revealed alterations in self-awareness in BPD which may hamper interpersonal functioning and may constitute a target for psychotherapeutic interventions.

In addition, it has to be mentioned that the task was an add-on experiment conducted after tasks that required the evaluation of self-referential information. Since BPD patients are more likely to experience higher levels of emotional arousal from such tasks as healthy participant, our findings may reflect state rather than trait alterations in BPD. Thus, future studies have to replicate our data in different contexts to further explore determinants of altered self-awareness in this clinical sample.

## Conclusions

In sum, our findings confirm alterations in self-awareness in BPD: Behavioral avoidance of cues that increase self-awareness was linked to a higher self-awareness in terms of a conscious decision to avoid these cues. The present study did not reveal the underlying mechanism or consequences for BPD patients’ social interactions. Nevertheless, one may assume that the observed alterations may disadvantageously affect social relations by restricting attentional resources available for the perception of social cues from others.
